# PM_2.5_ exposure on daily cardio-respiratory mortality in Lima, Peru, from 2010 to 2016

**DOI:** 10.1186/s12940-020-00618-6

**Published:** 2020-06-05

**Authors:** Vilma Tapia, Kyle Steenland, Bryan Vu, Yang Liu, Vanessa Vásquez, Gustavo F. Gonzales

**Affiliations:** 1 Faculty of Sciences and Philosophy, and Laboratory of Investigation and Development, Universidad Peruana Cayetano Heredia, Lima, Peru; 2 Department of Environmental Health, Rollins School of Public Health, Emory U, Atlanta, GA USA

**Keywords:** Air pollution, Particulate matter (PM_2.5_), Mortality, Lima

## Abstract

**Background:**

There have been no studies of air pollution and mortality in Lima, Peru. We evaluate whether daily environmental PM_2.5_ exposure is associated to respiratory and cardiovascular mortality in Lima during 2010 to 2016.

**Methods:**

We analyzed 86,970 deaths from respiratory and cardiovascular diseases in Lima from 2010 to 2016. Estimated daily PM_2.5_ was assigned based on district of residence. Poisson regression was used to estimate associations between daily district-level PM_2.5_ exposures and daily counts of deaths.

**Results:**

An increase in 10 μg/m^3^ PM_2.5_ on the day before was significantly associated with daily cardiorespiratory mortality (RR 1.029; 95% CI: 1.01–1.05) across all ages and in the age group over 65 (RR 1.04; 95% CI: 1.005–1.09) which included 74% of all deaths. We also observed associations with circulatory deaths for all age groups (RR 1.06; 95% CI: 1.01–1.11), and those over 65 (RR 1.06; 95% CI 1.00–1.12). A borderline significant trend was seen (RR 1.05; 95% CI 0.99–1.06; *p* = 0.10) for respiratory deaths in persons aged over 65. Trends were driven by the highest quintile of exposure.

**Conclusions:**

PM_2.5_ exposure is associated with daily cardiorespiratory mortality in Lima, especially for older people. Our data suggest that the existing limits on air pollution exposure are too high.

## Introduction

Approximately 4.2 million people are estimated to die annually from exposure to fine particles contained in polluted air [1]. Fine particulate matter (PM_2.5_) is considered the most harmful pollutant to human health, because it penetrates deep into the lungs [1]. Epidemiological studies conducted mainly in developing countries have associated PM_2.5_ with all cause and cause-specific mortality [2–7]. These studies have evaluated short-term and long-term PM_2.5_ exposure on mortality, primarily cardiovascular and respiratory deaths [3, 4, 8], but also some kinds of cancer [9–11]. Based on epidemiological evidence, the World Health Organization (WHO) estimated that air pollution is associated with premature deaths related to ischemic heart disease, strokes, chronic obstructive pulmonary disease, acute lower respiratory infections and lung cancer [2–4, 6, 7, 9].

Lima, the capital of Peru, is one of the most polluted cities in the Latin American region [12], with annual concentrations of PM_2.5_ ranging from 35 μg/m^3^ in the east side of Lima to 16 μg m^3^ in the districts within the center of Lima. The mean value for 2015 was 26 μg/m^3^ [13]. The air pollution mainly comes from vehicular emissions, although there is some contribution from industrial activity [14]. The government has implemented some regulations to reduce pollution and established safety thresholds for environmental concentrations [15]. Since then, PM_2.5_ levels have decreased markedly in the last 20 years, but high levels of pollutant are still observed in some areas of Lima [16, 17].

Gonzales and Steenland [18], analyzed PM_2.5_ data available from 2001 to 2010, and estimated that air pollution was responsible for 2300 premature deaths related to cardiorespiratory disease in adults, based on relative risk data from other parts of the world. Tapia et al. [19] have shown short-term effects of PM_2.5_ on emergency room visits in 9 Lima hospitals from 2010 to 2016. However, to date there is no specific epidemiological evidence that show the effects of air pollution on mortality in Peru. In this study, we have conducted daily time-series analysis using district-specific PM_2.5_ estimates in Lima [20] to evaluate whether daily environmental PM_2.5_ exposure is associated to respiratory and cardiovascular mortality in Lima during 2010 to 2016.

## Methods

### Study area

Lima is located on the central coast of Peru, at an average of 150 m above sea level, covers a geographical area of 2819 Km^2^ and a population density of 3392 inhabitants/km^2^. Lima has a population of 9,562,000 representing about 30% of the national population [21]. Lima is comprised of 43 districts and divided into four zones: North Lima, Central Lima, East Lima, and South Lima. We excluded 4 districts at high altitude (all above 570 m average altitude) due to uncertainty about the PM_2.5_ model predictions in these districts. The uncertainty was largely driven by the fact that ground monitoring stations providing inputs to the PM_2.5_ model were all located below 375 m, requiring a large extrapolation to these four high districts, using the model’s prediction of the altitude effect. These districts (district numbers 150,106, 150,107, 150,109, and 150,118) represented only 4% of the total population of Lima.

### Mortality data

Data on daily mortality were obtained from the Ministry of Health (MoH). Variables included in this data were age, gender, district of residence, district of occurrence of death, cause of death with respective International Classification of Disease 10th revision (ICD-10). Deaths from respiratory (J00-J99) and circulatory (I00-I99) disease were considered for the study. The database included 109,951 recorded deaths from respiratory and circulatory disease, between January 2010 and December 2016. We excluded four districts (10,228 deaths) because the model may be inaccurate above 375 m (as noted above), and also excluded some other observations because pollution on some days could not be estimated due to lack of satellite coverage (12,753 deaths) (see below); the remaining sample was 86,970.

### Meteorological and ambient PM_2.5_ data

Ground-monitoring PM_2.5_ data in Lima were available from March 2010 through December 2016, from 10 stations from the Servicio Nacional de Meteorología e Hidrología del Perú (SENAMHI, Ministry of the Environment), and 6 stations operated from 2011 to 2012 by Johns Hopkins University [22]. However, these data were not available on a daily basis during our study period, covering only about 10% of days. Hence, the ground-monitoring network was considered too sparse to adequately capture the spatiotemporal variability in PM_2.5_ levels that occurs in Lima. Thus, we based our PM_2.5_ exposure data from a model developed by Vu et al. [20]. Briefly, daily PM_2.5_ concentrations at a 1 km^2^ spatial resolution for 2010–2016 were estimated using a combination of the available ground measurements plus aerosol optical depth (AOD) data from satellites, and meteorological and land use data chemical transport models. AOD was obtained from NASA, using the MAIAC (Multi-Angle Implementation of Atmospheric Correction) algorithm. Meteorological fields (temperature, wind, and barometric pressure) were obtained from the European Centre for Medium-Range Weather Forecasts (ECMWF) and the Weather Research and Forecasting model coupled with Chemistry (WRF-Chem). A random forest model was used to regress the available ground measurements with 14 variables, including MAIAC AOD, meteorological variables from WRF-Chem and ECMWF, and land use variables. The overall cross-validation R^2^ value (and root mean square prediction error) was 0.70 (5.97 μg/m^3^), comparing predicted to observed ground level data. The mean difference between ground and predicted measurements was − 0.09 μg/m^3^. This regression model was then used to predict daily PM_2.5_ levels for each km^2^ grid across Lima. These estimates were then used in epidemiologic analyses, in which daily deaths were aggregated by district, and daily population-weighted average PM_2.5_ levels were calculated for each district from the 1 km^2^ data.

On every 16th day throughout the study period, we were unable to estimate PM_2.5_ due to lack of satellite coverage. Furthermore, PM_2.5_ estimates for October 15 to December 31, 2015 could not be made because the WRF-Chem model failed to estimate data within reasonable bounds for that period. Hence, we had PM_2.5_ estimates for 2236 days (91%) out of the 2465 days during the study period. The daily weather data (temperature and relative humidity) also was provided by SENAMHI.

The protocol was approved by Ethics Review Committee of Cayetano Heredia University (SIDISI code 202054).

### Statistical analysis

For each death we had the district of residence, which we used to assign PM_2.5_ daily exposure (or a lagged exposure) to that death. Using our daily estimates of PM_2.5_ at a 1 square km resolution, as well as estimated population in that same area, we created daily population-weighted PM_2.5_ averages by district, which were in turn assigned to all daily deaths in that district. Daily deaths in each district were grouped for a Poisson regression analysis.

We used generalized linear models with Poisson regression to estimate associations between daily district-level PM_2.5_ levels and daily counts of deaths for the outcomes of interest. PM_2.5_ effects were assessed using same day (lag 0), previous day (lag 1), 2 day (lag 2), 3 days (lag 3) average PM_2.5_ for two (0–2), 3 days (0–3) as well as the prior 30 days average; lag 1 was eventually chosen based on superior fit to the data using Akaike’s Information Criteria (AIC). To control for spatially varying factors and allow the analysis to be based on temporal contrasts only, the models included indicator variables for district to represent the geographical area over which deaths counts were spatially aggregated; this also controlled for spatial autocorrelation in the baseline deaths across the districts [23]. The models also included variables included for day of week, daily relative humidity, and maximum daily temperature. We compared two methods for controlling for long-term trends, either via parametric cubic splines with monthly knots, or with variables for month, year and an interaction between these variables (month*year). The latter fit appreciably better via the AIC and was used. The continuous PM_2.5_ variable was also categorized into quintiles: Q1st: 11.27–17.07 μg/m^3^; Q2nd: 17.08–18.60 μg/m^3^: Q3rd: μg/m^3^ 18.61–20.58), Q4th: 20.59–25.23 μg/m^3;^ and Q5th: 25.24–60.18 μg/m^3^. Mortality was analyzed as a whole, and also stratified by three age groups (< 18, 18–64, 65 years or more). We analyzed combined respiratory and circulatory deaths, and separately, respiratory deaths (ICD10 codes J00-J45), and circulatory mortality deaths (ICD10 codes I00-I99), as well as infectious respiratory disease (IRD)(ICD10 codes 00-J06, J09-J22) and cardiovascular disease (CVD)(ICD10 codes ICD10: I20-I22, I24, I25, I46-I50, I63-I67, I70, I73-I75, I77, I79, G45). Models for several other sub-categories with fewer daily deaths did not converge. Standard errors of coefficients were adjusted for over-dispersion, which generally was very modest. Analyses were conducted using SAS v9.4 PROC GENMOD (SAS Institute Inc., Cary, NC, USA).

## Results

The final analysis included 86,970 deaths; 59% were from respiratory causes, and 41% were from circulatory disease. The daily average was 22 deaths from respiratory disease and 15 from circulatory disease. On average, 74% of respiratory and circulatory deaths occurred in people over 65 years (Table 1).

**Table 1:** Descriptive characteristic of deaths, pollutant and meteorology data in Lima-Peru, during 2010 to 2016

Variable	Mean	SD	Median	25%	75%
Respiratory and circulatory deaths	37.2	7.29	37	32	42
Respiratory deaths	21.8	5.40	21	18	25
Circulatory disease	15.4	4.58	15	12	18
PM_2.5_ (*μg/m*^*3*^*)*	21.0	4.96	19.4	17.4	23.6
Temperature max (*°C*)	23.9	3.77	23.7	20.8	27.1
Relative humidity (%)	72.7	14.0	73.5	60.6	85.8

**Fig. 1:**
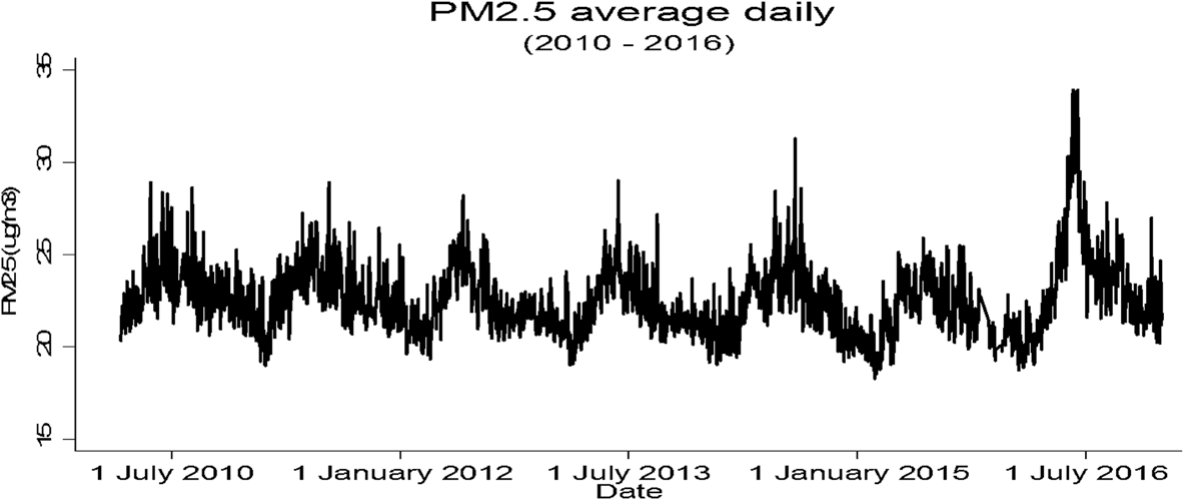
Daily average estimated PM2.5 in Lima, 2010–2016

The daily variation of estimated PM_2.5_ across Lima throughout the period is shown in Fig. 1. An increase in PM_2.5_ concentrations can be observed during the cold months (June-Sept). These values often exceeded the WHO air quality standards (24-h: 25 μg/m^3^), but were below the national Peruvian standard (24-h: 50 μg/m^3^).

The population-weighted average of PM2.5 estimated for Lima, across all districts during the study period, was 21.0 μg/m3. The highest average PM2.5 concentrations (29.3 ± 4.21 μg/m3) were observed in East Lima and the lowest average concentrations were found in Centre Lima (18.0 ± 1.95 μg/m3) (Table 2).

**Table 2:** Mean PM_2.5_ levels by district in Lima during 2010–2016

No.	District	X (μg/m^3^)	SD	No.	District	X (μg/m^3^)	SD
**North Lima** 22.7							
1	Ancon	22.3	2.43	5	Puente Piedra	27.2	2.93
2	Comas	27.3	3.31	6	San Martin de Porras	18.3	2.19
3	Independencia	23.3	2.43	7	Santa Rosa	21.1	1.98
4	Los Olivos	19.3	2.30				
**Centre Lima** 18.0							
1	Cercado de Lima	18.4	2.13	9	Miraflores	16.9	1.58
2	Barranco	16.8	1.47	10	Rimac	20.4	2.35
3	Breña	17.6	2.21	11	San Borja	19.7	2.27
4	Jesus María	16.5	2.31	12	San Isidro	17.0	1.97
5	La Victoria	19.2	2.25	13	San Luis	20.3	2.22
6	Lince	17.3	2.25	14	San Miguel	17.1	1.31
7	Magdalena	16.4	1.51	15	Santiago de Surco	20.3	1.79
8	Pueblo Libre	16.8	1.68	16	Surquillo	17.4	1.93
**South Lima** 20.5							
1	Chorrillos	17.9	1.25	7	San Bartolo	21.4	2.79
2	Lurin	18.6	1.39	8	San Juan de Miraflores	20.3	1.88
3	Pachacamac	27.7	1.55	9	Santa Maria	18.1	1.11
4	Pucusana	17.9	1.03	10	Villa El Salvador	19.4	1.95
5	Punta Hermosa	19.6	1.83	11	Villa Maria del Triunfo	24.7	2.14
6	Punta Negra	19.8	2.10				
**East Lima** 29.3							
1	Ate	29.1	4.12	4	San Juan de Lurigancho	32.1	4.92
2	El Agustino	27.4	3.77	5	Santa Anita	28.8	4.87
3	La Molina	29.1	3.38				

Air quality guidelines PM_2.5_: WHO: 10 μg/m^3^ annual mean; MINAM-Peru: 25 μg/m^3^ annual mean

Lag 1 PM_2.5_ levels generally produced the best fit models, and are presented here. Tables 3, 4 and 5 show the associations of daily PM_2.5_ with combined respiratory and circulatory deaths, and separate respiratory, and circulatory disease mortality; results are presented as rate ratios (RRs) and 95% confidence intervals (CIs) calculated for 10 μg/m3 increase in PM_2.5_ across all districts and years.

**Table 3:** Risk estimates and 95% confidence intervals (CI) for daily mortality for all cause (respiratory and circulatory deaths) for lag 1, 2010–2016

Age Group	*Β coefficient*	RR		*p*
		Exp(Β)*	95% CI	
**Respiratory and circulatory deaths (*n*** **= 86,970)**	0.0029	1.029*	1.005 1.052	0.012
Q1st (11.27–17.07)^a^	1.0			
Q2nd (17.08–18.60)	0.0254	1.025	1.006 1.044	0.007
Q3rd (18.61–20.58)	−0.0014	0.998	0.977 1.019	0.90
Q4th (20.59–25.23)	0.0129	1.012	0.987 1.038	0.31
Q5th (25.24–60.18)	0.0359	**1.036**	1.001 1.073	0.04
**Age: 65 or more(*n*** **= 64,792)**	0.0048	1.048*	1.005 1.093	0.028
Q1st (11.27–17.07)^a^	1.0	1.0		
Q2nd (17.08–18.60)	0.0303	1.030	0.997 1.065	0.07
Q3rd (18.61–20.58)	0.0228	1.023	0.985 1.061	0.25
Q4th (20.59–25.23)	0.0270	1.027	0.982 1.074	0.24
Q5th (25.24–60.18)	0.0788	**1.081**	1.014 1.153	0.016

^a^Referent category. Per unit of PM_2.5._ *Per 10 μg/m^3^ PM_2.5_. Model adjusted by day of week (dow), month, year, humidity and temperature

**Table 4:** Risk estimates and 95% confidence intervals (CI) for daily respiratory mortality for lag 1, 2010–2016

Age Group	*Β coefficient*	RR		*P*
		Exp(Β)*	95% CI	
**All deaths respiratory (*n*** **= 51,306)**	0.003	1.030*	0.990 1.073	0.15
Q1st (11.27–17.07)^a^	1.0	1.0		
Q2nd (17.08–18.60)	0.0271	1.027	0.993 1.062	0.12
Q3rd (18.61–20.58)	−0.0069	0.993	0.956 1.031	0.72
Q4th (20.59–25.23)	−0.0012	0.998	0.954 1.044	0.96
Q5th (25.24–60.18)	0.0256	1.025	0.963 1.091	0.42
**Age: 65 or more (38,293)**	0.0047	1.048*	0.999 1.098	0.06
Q1st (11.27–17.07)^a^	1.0	1.0		
Q2nd (17.08–18.60)	0.0246	1.024	0.986 1.064	0.22
Q3rd (18.61–20.58)	0.0042	1.004	0.961 1.048	0.85
Q4th (20.59–25.23)	0.0212	1.021	0.970 1.075	0.42
Q5th (25.24–60.18)	0.0515	1.052	0.979 1.131	0.16

^a^Referent category. Per unit of PM_2.5._ *Per 10 μg/m^3^ of PM_2.5_. Model adjusted by day of week (dow), month, year, humidity and temperature

**Table 5:** Risk estimates and 95% confidence intervals (CI) for daily circulatory mortality for lag 1, 2010–2016

Age Group	*Β coefficient*	RR		*p*
		Exp(Β)*	95% CI	
**All deaths circulatory (*n*** **= 35,664)**	0.0059	1.060*	1.008 1.113	0.02
Q1st (11.27–17.07)^a^	1.0	1.0		
Q2nd (17.08–18.60)	0.0386	1.039	0.999 1.081	0.054
Q3rd (18.61–20.58)	0.0074	1.007	0.963 1.053	0.74
Q4th (20.59–25.23)	0.0107	1.010	0.958 1.066	0.69
Q5th (25.24–60.18)	0.0849	**1.088**	1.009 1.174	0.03
**Age: 65 or more (*n*** **= 26,499)**	**0.0061**	1.062*	1.001 1.127	0.04
Q1st (11.27–17.07)^a^	1.0	1.0		
Q2nd (17.08–18.60)	0.0414	1.042	0.996 1.089	0.7
Q3rd (18.61–20.58)	0.0368	1.037	0.986 1.091	0.15
Q4th (20.59–25.23)	0.0352	1.035	0.974 1.101	0.26
Q5th (25.24–60.18)	**0.1055**	**1.111**	1.017 1.214	0.02
**CVD: all-age (32,937)**	0.0050	**1.051**	0.998 1.102	0.07
Q1st (11.27–17.07)^a^	1.0	1.0		
Q2nd (17.08–18.60)	0.0351	1.035	0.993 1.079	0.10
Q3rd (18.61–20.58)	0.0066	1.006	0.960 1.055	0.78
Q4th (20.59–25.23)	0.0194	1.019	0.963 1.079	0.50
Q5th (25.24–60.18)	0.0871	**1.091**	1.006 1.182	0.03
**CVD: age 65 or more (24,890)**	0.0041	1.041	0.979 1.107	0.20
Q1st (11.27–17.07)^a^	1.0	1.0		
Q2nd (17.08–18.60)	0.0376	1.038	0.990 1.088	0.12
Q3rd (18.61–20.58)	0.0379	1.039	0.984 1.095	0.16
Q4th (20.59–25.23)	0.0401	1.041	0.975 1.110	0.22
Q5th (25.24–60.18)	0.1015	**1.106**	1.008 1.215	0.03

^a^Referent category. Per unit of PM_2.5._ *Per 10 μg/m^3^ of PM_2.5_. Model adjusted by day of week (dow), month, year, humidity and temperature

PM_2.5_ was significantly positively associated with combined respiratory and circulatory deaths (RR 1.029; CI 95%: 1.005–1.052) and in the age group over 65 (74% of all deaths) (RR 1.048; CI 95%: 1.005–1.093) per 10 μg/m^3^ increase of PM_2.5_ (Table 3). Other age groups did not show positive trends. Positive trends were driven by the association in the top quintile, especially for deaths over 65 (RR 1.081, CI 95% 1.014–1.153).

A borderline significant RR (1.048; IC 95% 0.999–1.098; *p* = 0.06) for respiratory deaths in persons aged over 65 also observed (Table 4). We found significant positive RRs per 10 μg/m^3^ of increase in PM_*2.5*_ for circulatory deaths for all age groups (RR 1.06; CI 95%: 1.008–1.011), and for the group over 65 years (RR 1.06; IC 95% 1.001–1.127) (Table 5). Trends were driven by the top quintile with highest concentrations. Positive associations for CVD, a subset of circulatory disease, was seen in the group over 65 years (RR: 1.10; CI 95% 1.01–1.22) (Table 5). We could not do detailed analyses of other specific causes as deaths were too few and models did not converge.

## Discussion

In this study, we estimated PM_2.5_ data using a satellite-driven PM_2.5_ exposure model [20] which provided daily population-weighted average PM_2.5_ concentrations for all districts of Lima, from 2010 to 2016. We then examined PM_2.5_ short-term exposure in relation to cardiorespiratory mortality (ICD 10 I00-J99) in Lima, one of the most polluted cities in Latin America [12]. We observed positive associations of daily PM_2.5_ exposure with cardiorespiratory mortality, with an increase of 1.8% per 10 μg/m^3^ increase in PM_2.5_ concentration, driven largely by those over 65 years of age. Our results are congruent with previous findings of statistically associations of PM_2.5_ with combined respiratory and circulatory deaths [1, 3, 24]. In a systematic review of seven time series studies of all-cause deaths, the researchers found an increase of 1.4% per 10 μg/m^3^ increment in PM_2.5_ [24]. In another systematic review of 37 studies, the researchers reported a 0.9% increase in all-cause mortality per 10 μg/m^3^ increase in PM_2.5_ [2].

For circulatory mortality, our results show an increase of 3.7% per 10 μg/m^3^ increase in PM_2.5_ for both mortality for all ages, and 3.8% those over 65 years. We also found increased risk with CVD, a large subset of circulatory disease. Newell, in a systematic review of 91 published studies, found a 0.47% increase in cardiovascular mortality per 10 μg/m^3^ increase in PM_2.5_ [25]. Likewise, Zhao et al. observed an increased CVD mortality risk (0.68%) associated with a 10 μg/m_3_ increase in PM_2.5_ [7].

For respiratory mortality, we found an association only for the elderly group, which was borderline significant. Our results showed a slight increased risk of 3% per 10 μg/m^3^ increase of PM_2.5._ One systematic review reported positive effects, with a 10 μg/m^3^ increment in PM_2.5_ being associated with 1.51% (95% IC 1.01 to 2.01) in all cause respiratory mortality [24].

In our data adverse health effects were driven by high rate ratios in the 5th quintile. The exposure contrast between the 5^th^ and 4th quintile (mean 29.2 μg/m^3^ vs 22.6 μg/m^3^) was 6.6 μg/m^3^, larger than the inter-quartile range (IQR) of 6.2 μg/m^3^ for the entire population. Important exposure effects would have been lost with the use of broader categories in our categorical analyses.

Our study showed that PM_2.5_ exposure has a significant impact on the health of people in Lima, in particular among elderly people. The population structure is changing [21]; the number of individuals over 65 years is increasing. PM_2.5_ exposure along with a pre-existing condition, such as cardiometabolic disorders [26] or diabetes [27], has been shown to increase the risk of CVD deaths. Elderly people are more likely to have more of these conditions and be more vulnerable to the effects of air pollution.

Our study has several strengths. Firstly, we used a predictive model that estimated daily PM_2.5_ concentrations for 39 districts of Lima and for the entire study period, which replaced the relative scarce data from ground monitoring. Second, the allocation of PM_2.5_ concentrations to each death according to the district of residence allowed us to reduce possible exposure measurement by using district-specific PM_2.5_ estimates than using city-wide average data, thereby avoiding bias in our RR estimates. Third, we used the data of people who had habitual residence in Lima and died there. Limitations in our study included missing some exposure data (9% of days), some inaccuracy in our 1 km^2^ exposure estimates, and our inability to assign exposure estimates at a spatial resolution smaller than the district, due to lack of exact addresses. Another limitation is our lack of data regarding occupation, which will have led to some mismeasurement of estimated air pollution exposure for person who worked outside of their district.

Our study provides evidence regarding the effects of PM_2.5_ on cardio-respiratory deaths in moderate concentrations. The local recommendations for daily PM_2.5_ established by the Peruvian Ministry of Environment, are currently 25 μg/m^3^, compared to the recommendations given by WHO, which are 10 μg/m^3^. We believe that these recommendations for air quality in Peru are likely to be too high, and probably needs to be reviewed.

## Conclusions

The results of our study show that the short-term PM_2.5_ exposure is associated with all cause, respiratory and circulatory disease mortality, especially in the elder population. This result was observed at PM_2.5_ concentration, which is well below the daily limit established by the Ministry of Environment (MINAM in Spanish) in Lima.

## Data Availability

Data of this study is available upon request.

## Abbreviations

AOD: Aerosol optical depth
CI: Confidence interval
CVD: Cardiovascular disease
ECMWF: European Centre for Medium-Range Weather Forecasts
ICD: International Classification of Disease
IRD: Infectious respiratory disease
MAIAC: Multi-Angle Implementation of Atmospheric Correction
MINAM: Ministry of the Environment
MoH: Ministry of Health
PM2.5: Fine particulate matter
SENAMHI: Servicio Nacional de Meteorología e Hidrología del Perú
WHO: World Health Organization.
WRF-Chem: Weather Research and Forecasting model coupled with Chemistry
